# First Aid for the Hop-Picker

**Published:** 1924-09

**Authors:** A. H. Purcell Fox


### FIRST AID TO THE HOP-PICKER.

By the Rev. A. H. PURCELL FOX.

The annual exodus to the hop-gardens is just beginning, and hundreds of men, women and children are leaving the drabness and dirt of court and alley for the open air of the Garden of Kent. How do they fare for medical treatment? Many societies provide assistance of all kinds for these good folk, some sending skilled helpers and properly trained nurses. The district nurses in the hop-garden areas also afford valuable assistance during the season, and local hospitals and infirmaries always give a helping hand in case of emergencies.

### "Medical Service."

But a considerable proportion of the medical service rendered is of necessity allotted to people who, although not hospital-trained, have, at any rate, had some general instruction in the elements of physiology and first-aid, and perhaps, as in my own case, have served a term with the R.A.M.C. or a recognised society. It is quite useless to tell the "hopper" that you are not a doctor, for you will find yourself suddenly elevated to the much-coveted status in spite of all protests, and the promotion has its distinct drawbacks as well as its advantages. As a case in point I recall how I was besieged by a crowd one evening to attend a boy in the midst of it who had been badly injured in his play with another. Between the jangle of advice from the women and the surge of curiosity-mongers, it was difficult to discover what had happened. The injury promised to develop into serious complications, as the boy's eye had been badly hit with a rough piece of wood and was much contused. The organ itself proved on examination to be intact, but nobody would have cared to prophesy what might happen to it later. I urged the importance of skilled medical attention and disclaimed all responsibility if it were not obtained, but it was of no avail. Fortunately the case went straight forward without a hitch, and the father said good-naturedly upon my remonstrance to him, "Garn, guv'ner, a bloomin' doctor couldn't 'a done no more nor wot you 'ave!"

### A Homely Remedy.

The first casualty with which I had to deal gave me something of a shock. It was already a week old and had a "history." A little girl of five years of age had scalded her leg and foot, and there was a large blister covering the foot with several others down the leg, some of them broken. These were slightly septic and appeared to have gone scaly in the middle with a thick black crust. The wounds were not much inflamed and the general condition and temperature were satisfactory, but I was puzzled about the unusual appearance of the wounds. It turned out that the blackness was due to potato-peelings which had become imbedded after being laid on just as they were taken from the first dirty potato picked up! One needs to watch very carefully for any signs of infectious or contagious maladies, as there is a strong temptation to hide the victim, as well as ignorance concerning disease. As a result of our vigilance I was asked one Sunday morning by one of my assistants to go to a camp some five miles away to examine a suspect. I went and ferreted out the unhappy small boy who had had the misfortune to develop a rash. The rash was a familiar one, but it was not measles. There had been some sickness—for which I gave thanks—and the following represents fairly the conversation that I had with the mother.

"Where did you go yesterday?"

"To Deal, Sir."

"Did he have any fruit?"

"Yes, Sir, 'e 'ad some apples."

"Some! How many?"

"Don't know 'ow many, Sir, but a good few, cos they was given to 'im an' I couldn't stop 'im eatin' of 'em."

"Oh! What did he have for supper?"

"Two bloaters, Sir, an' I don't think they was very good."

Treatment—Castor oil.

### The Holiday Spirit.

Only the most compelling hindrances will prevent the hop-picker from going hopping, for hop-picking is not only a holiday, but a very lucrative one. You are told that "it sets the kids up for the winter" and the pay is sometimes used to buy winter footgear or clothing, although too often it is spent on far less worthy things. The result is that even "chronics" will manage to get through, for if granny is too infirm to pick, she is not too bad to sit in a chair somewhere and enjoy the fresh air. But the "chronics" are not the only or the worst anxiety. There are prospective mothers who will take great risks rather than be left behind, and you will sometimes get a sudden call and have to scurry around to find competant help for an impending birth. Occasionally somebody who is really ill will manage to conceal it, and it is not unusual to have a death in the camp.

### Dealing with Fresh Air.

When I first made acquaintance with the corrugated-iron huts in the hop-pickers' encampments, I asked my guide, who was the rector of the nearest village, how the pickers obtained air at night. The huts are not provided with windows and are very small. He replied, "Wait and see." When we got closer I perceived that every nook and cranny was carefully guarded against the invasion of fresh air by the simple method of plugging with straw and old garments. It is surprising to find any one alive in the morning when one remembers that a family of five will crowd into a hut about ten feet square! For those who have an opportunity to spend a holiday with the pickers there is an abundance of valuable experience to be had which can hardly be got in any other way. The fact that you have come all the way from London just to help them produces a response often lacking at other times, and when you have once gained the confidence of these people they are quite amenable to any simple hygienic teaching that you may tactfully impart to them.

